# Oleoylethanolamide supplementation enriches Akkermansia muciniphila and modulates intestinal barrier function in adults with obesity: A randomized, double-blind, placebo-controlled trial

**DOI:** 10.1080/29933935.2026.2622259

**Published:** 2026-02-21

**Authors:** Romeo Batacan, Amanda Rao, Yadav Sharma Bajagai, Dragana Stanley, David Briskey

**Affiliations:** a School of Health, Medical and Applied Sciences, Central Queensland University, Rockhampton, Australia; b RDC Clinical, Brisbane, Australia; c School of Human Movement and Nutrition Sciences, The University of Queensland, Brisbane, Australia

**Keywords:** Oleoylethanolamide, gut microbiome, occludin, *faecalibacterium prausnitzii*, *Akkermansia muciniphila*, intestinal barrier

## Abstract

Targeted modulation of the gut microbiome represents a promising nutritional strategy to support metabolic and intestinal health in overweight and obese adults. Oleoylethanolamide (OEA) is an endogenous lipid mediator that regulates satiety, lipid metabolism, and inflammation, but its effects on the human microbiome are not well defined. In this randomized, double-blind, placebo-controlled trial, 57 adults with obesity (BMI 30–40 kg/m²) received either 300 mg of TRPTI, providing 250 mg/day of OEA (*n* = 28), or placebo (*n* = 29) for 12 weeks. Outcomes included shotgun metagenomics, microbiome profiling, intestinal barrier and inflammatory biomarkers, and safety measures. OEA was safe and well-tolerated with no adverse changes in clinical biomarkers. Although overall microbial diversity remained stable, OEA induced selective, health-relevant compositional shifts. Notably, *Faecalibacterium prausnitzii* and *Akkermansia muciniphila* were enriched. These changes coincided with functional host benefits, including increased occludin at Week 12 and interleukin-2 at Week 6, while reducing interleukin-1β, consistent with improved epithelial barrier dynamics and reduced inflammation. Functional pathway analysis suggested enhanced microbial metabolic and redox capacity. These findings indicate OEA supplementation selectively enriches beneficial gut bacteria – particularly *A. muciniphila,* while improving gut barrier biomarkers and immune function without disrupting microbiome stability. These findings position OEA as a safe, targeted microbiome-modulating ingredient with potential applications for supporting gut and metabolic health.

## Introduction

Obesity represents one of the most pressing public health challenges of the 21 st century, affecting nearly 880 million adults worldwide^1^ and driving an escalating burden of metabolic comorbidities, including insulin resistance,^2^ nonalcoholic fatty liver disease (NAFLD),^3^ and cardiovascular disease.^4^ Beyond excessive adiposity, obesity is characterized by chronic low-grade inflammation.^5–7^ This inflammation originates from adipocyte dysfunction, immune cell infiltration, and dysregulated secretion of cytokines such as interleukin-6 (IL-6) and tumor necrosis factor-*α* (TNF-*α*).^7–9^ These processes exacerbate insulin resistance and tissue injury.^8–10^

Concurrently, the endocannabinoid system—an intricate network of lipid mediators, receptors, and metabolic enzymes—has emerged as a key regulator of appetite and metabolic homeostasis.^11^ Oleoylethanolamide (OEA), an endogenous fatty acid ethanolamide synthesized in the proximal small intestine during dietary fat absorption through enzymatic conversion of oleic acid, is also produced in neurons and adipose tissue.^12–14^ It influences gene expression related to fat absorption and fatty acid metabolism^15^^,^^16^ and it is present in small amounts (less than 2 µg/g) in foods like cocoa powder, oatmeal, and nuts.^17^

While OEA levels rise postprandially and fall during fasting, exogenous administration of OEA restores its concentration in metabolic disorders where its synthesis is impaired, such as obesity and accelerated aging associated with high-fat diets.^18^ OEA is primarily recognized for its capacity to induce satiety through high-affinity activation of the nuclear receptor peroxisome proliferator-activated receptor-*α* (PPAR-*α*), leading to reduced meal size, delayed meal initiation, and decreased meal frequency.^19^ These effects occur without engagement of psychoactive cannabinoid receptors.^20^

Beyond PPAR-*α*, OEA interacts with multiple targets, including the capsaicin receptor transient receptor potential vanilloid 1 (TrpV1) and orphan G protein-coupled receptors GPR55 and GPR119.^21^^,^^22^ Through these pathways, OEA enhances anandamide activity via competitive inhibition of fatty acid amide hydrolase (FAAH),^23^ modulates gut–brain neural circuits,^24^^,^^25^ induces anorexigenic hormones such as glucagon-like peptide-1 (GLP-1,^26^ suppresses hunger signals,^27^ and promotes lipid transport proteins such as cluster of differentiation 36 (CD36) and fatty acid transport protein (FATP).^18^^,^^28^ In vitro and animal studies demonstrate OEA’s ability to enhance lipid metabolism,^18^ improve insulin and cholesterol profiles,^29^^,^^30^ and activate feeding-related neural networks.^27^^,^^31^^,^^32^

Importantly, OEA exhibits protective effects across a spectrum of metabolic, gastrointestinal, neurodegenerative, and inflammatory conditions.^13^^,^^33^^,^^34^ It downregulates the NF-κB proinflammatory pathway,^35^ reduces oxidative stress markers such as malondialdehyde (MDA), and attenuates nociceptive responses, underscoring its anti-inflammatory and antioxidant properties.^36^ Exogenous OEA supplementation in obese patients with NAFLD (250 mg/day for 8 to 12 weeks) has yielded significant improvements in triglycerides, fasting blood glucose, C-reactive protein, IL-6, TNF-*α*, and HDL-C levels. These findings highlight its therapeutic potential in cardiometabolic disorders.^37^

Emerging data link OEA’s systemic actions to modulation of the gut microbiome. Sub-chronic OEA treatment in rodents shifts fecal microbial communities toward a lean-like phenotype by decreasing the Firmicutes:Bacteroidetes ratio and reducing proinflammatory taxa.^38^ In Caco-2 cell models, OEA restores epithelial barrier function by reducing hyperpermeability and inflammation.^39^ In obese adults, OEA supplementation increases the abundance of *Akkermansia muciniphila*, a mucin-degrading bacterium associated with enhanced fat oxidation, strengthened gut barrier integrity, and immunomodulation, while decreasing carbohydrate intake.^40^^,^^41^ OEA may also modulate these effects by promoting the expansion of *A. muciniphila* levels in the body through the activation of GPR119 and PPAR-*α* receptors. These findings suggest that OEA’s metabolic and anti-inflammatory benefits may be mediated, in part, by shaping microbial communities.

In addition to human studies, a substantial body of preclinical evidence supports OEA’s metabolic and microbiota-related actions in obesity. Recent work by Cimmino et al. (2025) demonstrated that, in rodent models of diet-induced obesity, OEA does not broadly restructure gut microbial communities but selectively increases the relative abundance of *A. muciniphila*, aligning with human observations.^42^ An earlier study by Izzo and colleagues further showed that peripheral OEA levels are dysregulated in obese rodents, with both intestinal and systemic concentrations reduced compared with lean controls.^43^ These findings suggest that impaired endogenous OEA signaling may contribute to obesity-related metabolic dysfunction and highlight the relevance of restoring OEA tone through exogenous supplementation.

The global rise in obesity has led to widespread inflammatory and metabolic complications, highlighting an urgent need for novel, safe, and targeted interventions. Although preclinical and early clinical findings suggest that OEA may offer such benefits,^33^^,^^44^ no placebo-controlled trial has directly examined whether OEA supplementation can alter the gut microbiota in adults with obesity. Therefore, we designed a randomized, double-blind study to compare daily oral OEA versus placebo in obese participants, with the primary aim of assessing changes in microbial diversity and community structure. It was hypothesized that supplementation with OEA would modulate circulating biomarkers and the gut microbiome, leading to improvements in gut barrier function, reduced inflammation and enhanced quality of life compared with a placebo. Specifically, we evaluated whether OEA selectively enriches beneficial taxa, such as *A. muciniphila,* and whether these microbiome shifts coincide with reductions in systemic inflammation and improvements in metabolic markers.

## Methods

### Participants

The study enrolled 57 individuals from Australia, and 44 of them completed all study procedures. Participants included both males and females aged between 23 and 64 years. Eligibility criteria required individuals to be between 18 and 65 years of age with a body mass index (BMI) ranging from 30 to 40 kg/m². Those with ongoing or serious health conditions, recent illness, use of certain medications or gut-altering supplements, active smoking, regular alcohol consumption, or allergies to study ingredients were excluded. Additional exclusions applied to pregnant or lactating women, females of childbearing potential, individuals using medications that affect immune or inflammatory responses, recent participants in similar research, and those with a history of cancer, HIV, or chronic steroid use.

### Study groups

Participants were randomly assigned to one of two study arms using Random Allocation Software (Sealedenvelope.com.au), with both participants and investigators blinded to group allocation. The intervention group (*n* = 28) received TRPTI brand bioavailable oleoylethanolamide (OEA) powered by LipiSperse Technology. Each participant consumed two 150 mg capsules of TRPTI daily, one in the morning and one in the evening, for a total daily dose of 300 mg. Each TRPTI capsule contained no less than 125 mg of OEA, for a total daily dose of OEA of no less than 250 mg.

The control group (*n* = 29) received a placebo consisting of microcrystalline cellulose (MCC), encapsulated in opaque capsules identical in appearance to those used in the OEA group. The placebo was manufactured by a Brisbane-based compounding pharmacy in accordance with Good Manufacturing Practice (GMP) standards. Participants in the placebo group followed the same dosing schedule as the intervention group, taking one capsule in the morning and one in the evening.

### Study protocol

This study adhered to international and national standards, including the International Conference on Harmonization (ICH) Good Clinical Practice (GCP) guidelines, the Therapeutic Goods Administration (TGA) Note for Guidance on GCP, and ethical guidelines outlined in Additional Ethical Considerations. It employed a randomized, double-blind, placebo-controlled design. Ethical approval was obtained from the Bellberry Human Research Ethics Committee (approval number: 2020-07-675), and the study was registered with the Australian New Zealand Clinical Trials Registry (ACTRN12621000017820).

Recruitment was carried out through a combination of public media announcements and existing participant databases. Interested individuals were assessed for eligibility based on predetermined criteria and were thoroughly briefed about the study procedures and expectations. Those who met the inclusion criteria provided written, electronic consent via Smartwaver software before formally entering the study. Once enrolled, participants were randomly allocated to receive either OEA or placebo capsules. Each participant was given a sealed, opaque bottle containing capsules that were indistinguishable in appearance, ensuring blinding was maintained across both arms.

Before beginning the intervention, participants completed a series of baseline assessments. These included blood collection, a liver stiffness evaluation using FibroScan, anthropometric measurements (including height, body weight, waist circumference, hip circumference, systolic and diastolic blood pressure), stool sample collection, and completion of validated questionnaires: 24-hour dietary recall, SF-36 (Quality of Life), Perceived Stress Scale (PSS), and the Pittsburgh Sleep Quality Index (PSQI-10).

Participants were instructed to collect stool samples at home using the OMR-200 kit (DNA Genotek, Canada), which they received during their initial visit, along with instructions. The OMR-200 kit preserves microbial DNA at ambient temperature. In accordance with manufacturer specifications, samples were stored at room temperature for up to 7 days following collection, after which they were returned to the clinic and transferred to ultra-low temperature freezers (–80 °C) for long-term storage prior to analysis. Temporary storage at chilled temperatures (4 °C to –20 °C) was permitted after collection and during transport and is not known to affect sample stability in this collection system. Subsequent analysis included assessments of microbiota composition, metagenomic profiles, calprotectin levels, and short-chain fatty acids (SCFAs) concentrations.

Venous blood was drawn from the antecubital region into both serum and EDTA tubes (BD, Australia). Plasma from the EDTA tubes was processed immediately through centrifugation at 4 °C (2,100 × g for 10 minutes). Serum samples were allowed to clot for 30 minutes prior to centrifugation. Processed serum and plasma were aliquoted and stored at –80 °C until analysis.

A comprehensive biomarker panel was analyzed in plasma or serum, including enzyme/liver function tests (E/LFT), high-sensitivity C-reactive protein (hsCRP), homocysteine, fatty acid binding protein (FABP), glucagon-like peptide-1 (GLP-1), gastric inhibitory polypeptide (GIP), glutathione S-transferase (GST), interferon gamma (IFN-*γ*), interleukin-1 beta (IL-1β), interleukin-2 (IL-2), monocyte chemoattractant protein-2 (MCP-2), mucin 2 (MUC2), occludin, lipopolysaccharide (LPS), claudin-1 (CLDN-1), transforming growth factor beta (TGF-*β*), tumor necrosis factor alpha (TNF-*α*), and zonulin.

E/LFT, hsCRP, and homocysteine were analyzed using a Biobase clinical chemistry analyzer (Biobase, China) with assay kits and calibrators supplied by the manufacturer. Pro-inflammatory cytokines (IFN-*γ*, TNF-*α*, IL-2, and IL-1β) were measured using a bead-based multiplex immunoassay platform (Milliplex®, MilliporeSigma) according to the manufacturer’s instructions. Glutathione S-transferase (GST) was quantified using colorimetric assay kits (Sigma-Aldrich, USA) on a clinical chemistry analyzer. All remaining biomarkers (FABP, GLP-1, GIP, MCP-2, MUC2, occludin, LPS, CLDN-1, TGF-*β*, and zonulin) were measured by commercially available ELIZA kits (Thermo Fisher Scientific, Australia) following the manufacturers’ protocols.

After all baseline data were collected, participants began taking the study product for a total of 12 weeks. Throughout the intervention period, they were contacted regularly to ensure adherence, address any concerns, and monitor for any adverse effects. Complete assessments (matching the baseline measures) were conducted again at week 6 (midpoint) and at the end of the 12-week trial.

### Statistical analysis

Sample size requirements were estimated based on data reported by Depommier et al. (2019) in a randomized clinical trial of pasteurized/live *A. muciniphila* supplementation in overweight and obese adults.^45^ In that study, the mean baseline abundance of *A. muciniphila* was approximately 4.5 log₁₀ copies/g feces (SD ≈ 0.21). A 20% increase corresponds to ~0.18 log units. Assuming a residual standard deviation of 0.21 and targeting a two-sided *α* = 0.05 with 80% power, this effect size translates to Cohen’s d ≈ 0.84. Accordingly, a minimum of 22 participants per group is required to achieve sufficient statistical power to detect a 20% change in *A. muciniphila* abundance. This calculation provides a conservative estimate consistent with previous human intervention studies demonstrating substantial modulation of *A. muciniphila* abundance. To account for an anticipated dropout rate of approximately 45%, up to 40 participants were recruited into each group. Statistical analysis began with an assessment of data normality for each variable. Depending on the distribution, either a *t*-test was applied for normally distributed data or a Wilcoxon test for non-normally distributed data to compare outcomes between the two groups.

Statistical significance was assessed using two complementary thresholds depending on the analysis performed. For analyzes involving multiple simultaneous hypothesis testing, including microbiome differential abundance analyzes, *p*-values were also adjusted for multiple comparisons using the Benjamini–Hochberg false discovery rate (FDR) procedure, and results were considered statistically significant at pre-adjusted *p* < 0.05 and post-adjusted *q* < 0.05. For analyzes not subject to multiple testing, including predefined primary outcomes and targeted biomarker analyzes, statistical significance was defined as *p* < 0.05.

### Shotgun metagenomic sequencing

DNA was extracted from fecal samples using EconoSpin All-In-One DNA Only Mini Spin Column (Epoch Life Science, Sugar Land, TX, USA), and its quality was assessed using the Qubit 3.0 fluorometer (Thermo Fisher, Waltham, MA, USA) and agarose gel electrophoresis. The shotgun metagenomic sequencing was outsourced. Next-generation sequencing libraries were prepared using the VAHTS Universal Plus DNA Library Prep Kit (Vazyme, Nanjing, China) for Illumina V2, following the manufacturer’s protocol. Genomic DNA (200 ng per sample) was enzymatically fragmented to <500 bp and subjected to end repair, phosphorylation, and dA-tailing in a single reaction. Adapters were ligated by T–A ligation, and adapter-ligated fragments were size-selected with VAHTS DNA Clean Beads (Vazyme) to yield ~410 bp fragments (insert size ~350 bp). Libraries were amplified for 6–8 PCR cycles using P5 and indexed P7 primers, purified with VAHTS DNA Clean Beads, and quantified with a Qubit 3.0 Fluorometer (Invitrogen, CA, USA). Indexed libraries were pooled, multiplexed, and sequenced on an Illumina NovaSeq 6000 platform (Illumina, San Diego, CA, USA) using a 2 × 150 bp paired-end configuration. Image analysis and base calling were performed with NovaSeq Control Software (NVCS) and RTA 3. Demultiplexing was performed with bcl2fastq v2.20. Raw reads were filtered to remove: (i) read pairs containing adapter sequences, (ii) read pairs in which >10% of bases were undetermined (*N*), and (iii) read pairs in which > 50% of bases had a Phred quality score <20.

### Sequence quality control and preprocessing

Data integrity was confirmed by generating MD5 checksums.^46^ Sequence quality was assessed with FastQC v0.11.2,^47^ and summary reports were compiled with MultiQC v1.11.^48^ Reads were initially filtered and trimmed with fastp to remove low-quality sequences, reads <50 bp, and residual adapter contamination.

Further quality refinement was carried out using KneadData v0.7.10, which used Trimmomatic^49^ to trim bases with Phred scores <3 from read ends and apply a sliding window filter (4:15) to remove low-quality regions. Host-derived (human) DNA sequences were removed by aligning sequences to the human reference genome and discarding matching reads. Forward and reverse reads were subsequently merged. Following quality control and host depletion, each sample retained an average of 38.1 ± 4.4 million high-quality reads (mean ± SD), with a minimum Phred score of 35 and <1% overrepresented sequences.

### Taxonomic and functional analysis

Biomarker taxa distinguishing treatment groups at baseline and week 12 were identified using Linear Discriminant Analysis Effect Size (LEfSe). To assess whether between-group differences in important selected taxa at week 12 represented treatment-induced changes, linear mixed-effects models with time × treatment interaction terms were performed, including subject as a random effect to account for repeated measures. Analysis of covariance (ANCOVA) with baseline adjustment was used to compare week 12 abundances while controlling for baseline values.

Trimmed and quality-filtered sequences were analyzed using HUMAnN v3.0^50^ Taxonomic composition was profiled through the MetaPhlan3 with the ChocoPhlAn marker gene database, while functional potential was inferred using the UniRef90 protein database.^51^ Downstream statistical analyzes and data visualization were conducted in R using the *phyloseq,*^52^ *vegan,*^53^ and *microeco*^54^ packages.

### Fecal SCFAs and calprotectin

SCFAs were analyzed following a protocol adapted from Tao et al.^54^ Fecal samples were first acidified with sulfuric acid and then extracted with ethyl ether. The extracted SCFAs were separated and quantified using a Shimadzu gas chromatograph equipped with a flame ionization detector (GC-FID).

For fecal calprotectin analysis, samples were dehydrated using a centrifugal vacuum dryer, and dry weights were recorded. The dried samples were then reconstituted in assay buffer and analyzed using a calprotectin ELIZA kit (Epitope Diagnostics) according to the manufacturer’s instructions. Absorbance readings were obtained using a Biobase BL-EK-10C microplate reader.

### Fibroscan

Liver assessments were conducted using transient elastography (FibroScan) at a local imaging center (I-MED Radiology, Brisbane, Australia) by a qualified radiographer. The FibroScan device measured shear wave velocity to assess liver stiffness, providing a non-invasive evaluation of hepatic tissue for signs of fibrosis or steatosis.

## Results

### Demographics

Fifty-seven participants were enrolled in the study, with 44 completing all study requirements (Table 1a). Both groups were well matched at baseline, with no significant differences between the groups for any baseline measures (Table 1b).

**Table 1a.** Baseline demographics.

	OEA (*n* = 22)	Placebo (*n* = 22)
Height (m)	1.69 (0.10)	1.66 (0.06)
Weight (kg)	100.29 (13.57)	97.07 (10.37)
BMI (kg/m^2^)	35.00 (2.62)	35.10 (2.74)
Waist circumference (cm)	102.99 (10.15)	101.63 (8.01)
Hips circumference (cm)	121.20 (7.57)	120.70 (6.92)
Systolic BP (mmHg)	129.13 (11.80)	125.52 (12.48)
Diastolic BP (mmHg)	87.29 (5.45)	83.60 (6.55)

Data shown as mean (SD).

**Table 1b.** Participant anthropometrics and change from baseline to week 12 for sub-groups (>35 BMI and <35 BMI).

BMI <35	OEA Baseline	OEA Change W12	Placebo Baseline	Placebo Change W12	
Weight (kg)	93.8 (14.34)	−1.56 (3.72)*	93.98 (6.96)	1.16 (1.96)	
BMI (kg/m2)	33.00 (1.38)	−0.42 (1.35)	32.84 (1.36)	0.26 (0.83)	
Waist circumference (cm)	99.39 (12.26)	−0.73 (6.37)	100.40 (6.96)	1.22 (2.54)	
Hips circumference (cm)	115.38 (3.16)	−1.3 (0.14)^#^	117.52 (3.78)	0.13 (2.32)	

BMI >35	OEA Baseline	OEA Change W12	Placebo Baseline		Placebo Change W12
Weight (kg)	101.99 (13.25)	0.41 (2.11)	103.39 (10.92)		−0.40 (3.10)
BMI (kg/m2)	37.30 (1.95)	0.15 (0.08)	37.56 (1.75)		−0.15 (1.15)
Waist circumference (cm)	104.38 (9.02)	1.63 (2.63)	104.06 (9.03)		−0.46 (4.57)
Hips circumference (cm)	127.05 (7.12)	0.97 (3.68)	124.16 (8.25)		−1.01 (3.97)

*p < 0.05 compared to the same time point of the placebo group. ^#^trending toward significance; (*p* = < 0.01). Data is presented as mean (SD).

### Biochemistry

Following 12 weeks of supplementation, there was no significant difference between groups in the absolute concentration of any of the pathology markers analyzed (Table 2).

**Table 2.** Pathology marker outcomes.

	OEA			Placebo		
Parameter	Baseline	Week 6	Week 12	Baseline	Week 6	Week 12
FABP (ng/mL)	7.67 (14.06)	8.11 (15.01)	8.3 (14.61)	10.57 (35.41)	9.21 (24.98)	8.69 (21.83)
MCP2 (pg/mL)	6.00 (4.02)	6.26 (3.45)	6.18 (3.15)	6.48 (3.51)	6.7 (4.55)	5.95 (3.8)
GLP-1 (pg/mL)	336.10 (289.29)	346.94 (312.68)	437.01 (379.69)	502.02 (463.84)	475.38 (335.88)	461.86 (334.36)
GIP (pg/mL)	4.01 (2.12)	4.31 (2.38)	4.26 (2.34)	6.17 (5.83)	5.43 (5.51)	6.12 (5.79)
OCLN (pg/mL)	19.04 (40.34)	22.93 (46.04)	22.52 (40.5)	13.2 (15.9)	14.56 (18.87)	8.08 (16.37)
Zonulin (ng/mL)	0.03 (0.08)	0.02 (0.04)	0.07 (0.21)	0.12 (0.26)	0.09 (0.21)	0.09 (0.19)
CLDN-1 (ng/mL)	0.78 (1.11)	0.74 (1.09)	0.82 (1.05)	0.97 (0.84)	0.74 (0.69)	0.77 (0.78)
MUC2 (ng/mL)	12.8 (8.18)	13.13 (8.8)	13.31 (8.3)	15.3 (10.35)	13.13 (7.89)	14.16 (8.53)
IFN-*γ* (pg/mL)	23.88 (21.37)	26.06 (26.66)	23.24 (26.02)	22.47 (20.08)	23.98 (21.7)	32.75 (38.09)
GST (ng/mL)	0.92 (0.83)	0.86 (0.82)	0.93 (0.83)	0.65 (0.48)	0.62 (0.46)	0.69 (0.46)
TGF-*β* (pg/mL)	2091.17 (653.56)	2026.96 (762.06)	2035.07 (851.98)	2241.02 (887.84)	2093.82 (903.34)	2193.68 (969.37)
IL1-*β* (pg/mL)	147.91 (74.33)	130.04 (78.97)	148.84 (77.72)	119.33 (65.09)	140.43 (78.08)	129.98 (61.79)
Endotoxin (ng/mL)	89.13 (30.6)	78.98 (38.71)	82.54 (29.52)	89.51 (52.69)	69.57 (42.11)	89.03 (36.18)
IL-2 (pg/mL)	11.92 (38.9)	13.91 (41.32)	12.38 (37.94)	12.82 (18.71)	12.33 (17.68)	12.23 (18.84)
TNF-*α* (pg/mL)	6.18 (28.49)	6.84 (31.69)	7.38 (32.98)	14.32 (38.25)	14.3 (36.14)	16.47 (39.77)

Data is presented as mean (SD); *p < 0.05 compared to the same time point of the placebo group. CLDN-1, Claudin 1; FABP, fatty acid binding protein; GIP, gastric inhibitory polypeptide; GLP-1, glucagon-like peptide 1; GST, glutathione s-transferase; IFN-γ, interferon gamma; IL1-β, interleukin 1 beta; IL-2, interleukin 2; MCP-2, monocyte chemoattractant protein 2; MUC2, Mucin 2; OCLN, Occludin; TGF-β, transforming growth factor beta 1; TNF-α, tumour necrosis factor alpha.

Analysis of the mean change from baseline to Week 6 and Week 12 revealed significant differences in several outcome measures (Table 3). OEA supplementation significantly increased occludin (OCLN) levels at Week 12 and interleukin-2 (IL-2) levels at Week 6 compared to the placebo group. Interleukin-1β (IL-1β) levels were significantly reduced in the OEA group at Week 6 compared to the placebo and at Week 12 compared to baseline. The GLP-1 concentration increased in the OEA group at week 6 and week 12, which was trending toward significance (*p* < 0.1). In contrast, the placebo group showed no significant changes in any markers, with a general downward trend over time. No other significant between-group differences were observed.

**Table 3.** Change from baseline pathology marker outcomes.

	OEA		Placebo	
	Week 6	Week 12	Week 6	Week 12
FABP (ng/mL)	0.01 (3.26)	−0.07 (3.12)	−2.19 (11.99)	−3.69 (17.73)
MCP2 (pg/mL)	0.37 (2.26)	−0.11 (2.67)	0.15 (2.97)	−0.03 (2.1)
GLP-1 (pg/mL)	81.36 (341.42)^#^	100.91 (375.21)^#^	−55.69 (262.60)	−39.92 (282.20)
GIP (pg/mL)	0.28 (0.9)	0.26 (0.66)	−0.5 (1.26)	−0.28 (1.37)
OCLN (pg/mL)	2.17 (13.18)	2.65 (7.89)*	1.00 (11.74)	−4.36 (11.34)
Zonulin (ng/mL)	0.01 (0.06)	0.05 (0.22)	−0.04 (0.09)	−0.06 (0.13)
CLDN-1 (ng/mL)	−0.08 (0.33)	0.01 (0.55)	−0.27 (0.53)	−0.18 (0.63)
MUC2 (ng/mL)	0.4 (4.28)	0.15 (4.95)	−2.24 (8.47)	−1.92 (6.48)
IFN-γ (pg/mL)	1.11 (16.29)	0.08 (20.9)	2.1 (8.31)	9.16 (23.38)
GST (ng/mL)	−0.07 (0.26)	−0.03 (0.26)	−0.02 (0.15)	−0.03 (0.16)
TGF-b (pg/mL)	9.77 (653.21)	−87.83 (1088.02)	−134.87 (861.9)	27.11 (948.99)
IL-1b (pg/mL)	−15.96 (59.91)*	−5.00 (28.72)	13.92 (34.55)	3.58 (28.18)
Endotoxin (ng/mL)	-9.28 (37.73)	−6.59 (37.62)	−18.93 (39.77)	−12.69 (47.93)
IL-2 (pg/mL)	1.27 (3.04)*	−0.21 (5.42)	−1.14 (3.81)	−2.33 (3.83)
TNF-a (pg/mL)	0.11 (2.58)	0.02 (2.85)	−1.17 (7.)	−1.94 (10.56)

Data is presented as mean (SD); *p < 0.05 compared to the same time point of the placebo group, ^#^*p* < 0.1 compared to the same time point of the placebo group.CLDN-1, Claudin 1; FABP, fatty acid binding protein; GIP, gastric inhibitory polypeptide; GLP-1, glucagon-like peptide 1; GST, glutathione s-transferase; IFN-γ, interferon gamma; IL1-β, interleukin 1 beta; IL-2, interleukin 2; MCP-2, monocyte chemoattractant protein 2; MUC2, Mucin 2; OCLN, Occludin; TGF-β, transforming growth factor beta 1; TNF-α, tumour necrosis factor alpha.

No significant between-group differences were observed in the general safety pathology markers (Table 4).

**Table 4.** Safety pathology marker outcomes.

	OEA			Placebo			
	Baseline	Week 12	change	Baseline	Week 12	change	*p*-values
Albumin (g/L)	44.73 (2.35)	44.25 (2.85)	−2.3 (9.85)	44.24 (1.64)	44.06 (1.9)	−0.07 (1.59)	0.30
ALT (U/L)	14.34 (9.71)	15.9 (10.94)	2.62 (7.96)	24.44 (27.77)	18.3 (18.)	−5.13 (25.67)	0.19
AST (U/L)	23.85 (10.5)	24.17 (10.99)	1.14 (9.79)	37.17 (30.88)	29.55 (30.24)	−10.8 (23.87)	0.04
CHO (mmol/L	5.99 (0.84)	6.22 (1.23)	−0.1 (1.79)	5.62 (0.98)	5.58 (0.99)	0.04 (0.9)	0.82
Homocysteine (umol/L)	12.34 (4.17)	12.76 (3.44)	−0.15 (5.93)	12.62 (4.5)	11.69 (3.69)	−1.24 (5.93)	0.54
HDL-C (mmol/L)	1.19 (0.4)	1.87 (3.21)	0.6 (3.04)	1.25 (0.28)	1.25 (0.26)	0.04 (0.17)	0.38
TG (mmol/L)	1.7 (1.11)	2.01 (2.41)	0.2 (1.57)	1.61 (0.86)	1.42 (0.61)	−0.24 (0.53)	0.23
Total Protein (g/L)	66.79 (4.41)	68.02 (13.13)	1.42 (11.7)	65.14 (5.21)	64.51 (3.27)	−0.25 (4.62)	0.54
GGT (U/L)	35.12 (22.64)	32.09 (16.35)	−4. (14.97)	51.62 (45.99)	44.68 (38.7)	1.7 (38.57)	0.52
Tbil (umol/L)	10.12 (5.12)	10.48 (4.28)	−0.34 (5.69)	11.36 (8.44)	8.9 (4.81)	−3.73 (8.86)	0.14
Creatinine (umol/L)	70.87 (16.)	68.02 (14.45)	−2.46 (9.97)	69.06 (22.53)	71.86 (25.67)	6.39 (26.3)	0.16
Glucose (mmol/L)	5.12 (1.44)	4.86 (1.02)	−0.34 (1.01)	4.88 (0.81)	4.93 (0.84)	0.03 (0.64)	0.16
hsCRP (mg/L)	6.33 (4.89)	6.65 (4.74)	−0.43 (3.14)	5.77 (3.66)	6.94 (4.53)	1.22 (3.49)	0.10
RBC GST (uM)	1999.5 (351.2)	2169.6 (433.2)	182.4 (382.7)	2050.2 (360.0)	2080.5 (522.0)	10.6 (393.7)	0.16
Plasma GST (uM)	2.1 (0.62)	2.26 (1.02)	0.16 (1.06)	2.45 (0.83)	2.72 (0.99)	0.21 (1.02)	0.89

Data is presented as mean (SD). *p < 0.05 compared to the same time point of the placebo group. ALT, alanine transaminase; AST, aspartate aminotransferase; CHO, cholesterol; GGT, gamma-glutamyl transferase; GST, glutathione S-transferase; HDL-C, high density lipoprotein cholesterol; hsCRP, high sensitivity C-reactive protein; RBC, red blood cell; Tbil, total bilirubin; TG, triglycerides.

### Taxonomy

#### Alpha diversity and microbiota richness

**Figure 1.**
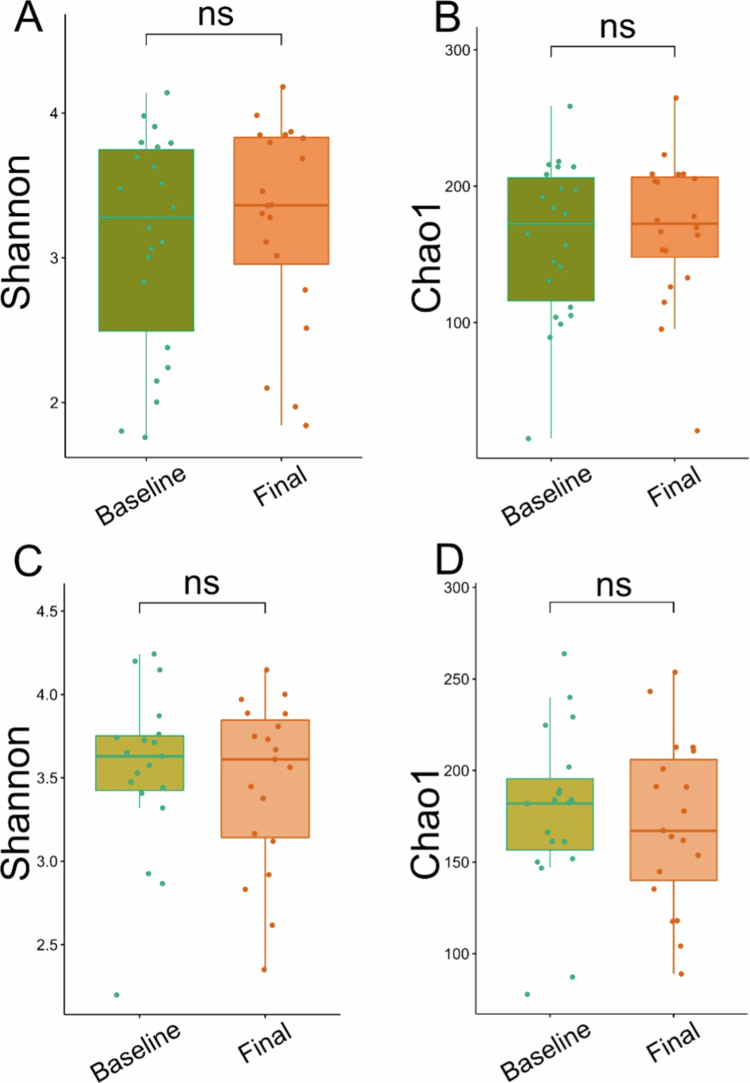
Alpha diversity and microbiota richness of the two groups. A = Alpha diversity in the OEA group as measured with the Shannon diversity index. B = Microbiota richness in the OEA group as measured with the Chao1 index. C = Alpha diversity in the Placebo group as measured with the Shannon diversity index. D = Microbiota richness in the Placebo group as measured with the Chao1 index.

No differences in alpha diversity or microbiota richness were observed between groups, as assessed by the Shannon and Chao1 indices (*p* > 0.05; Figure 1A−D).

#### Beta diversity and overall microbiota profile

**Figure 2.**
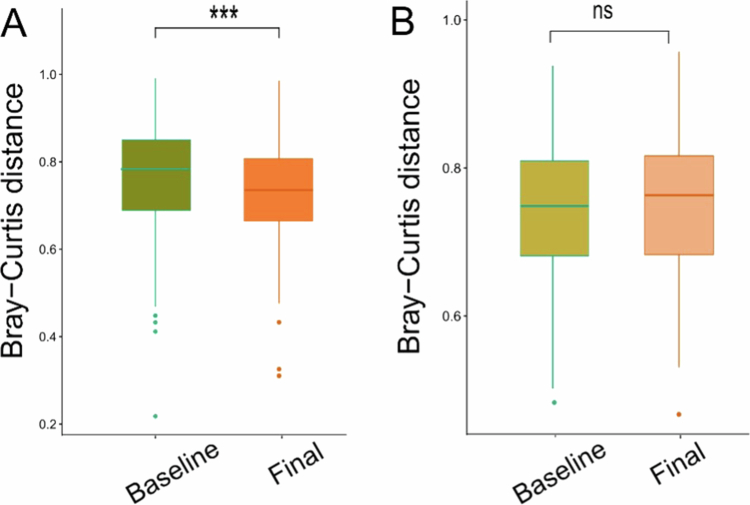
Comparison of Bray–Curtis dissimilarity between Baseline and Final time points. Distances were calculated based on relative abundances of microbial taxa and are displayed as boxplots. Lower values indicate greater similarity in community structure, while higher values indicate divergence. A = OEA group; B = Placebo group.

PERMANOVA of Bray–Curtis distances revealed no significant differences in overall microbial community composition for any of the following comparisons: (i) baseline vs. final time point within the OEA group, (ii) baseline vs. final time point within the placebo group, (iii) OEA vs. placebo at baseline, and (iv) OEA vs. placebo at the final time point. Consistently, PCoA ordination of Bray–Curtis distances showed substantial overlap among groups (Supplementary Fig. S1). However, analysis of within-group Bray-Curtis distance revealed significantly greater variability in the OEA group at baseline compared with the final time point, whereas the placebo group showed no change (Figure 2A−B). In addition, within-group Bray–Curtis distances were significantly higher in the OEA group than in the placebo group at baseline, whereas no such differences were observed at the final time point.

**Figure 3.**
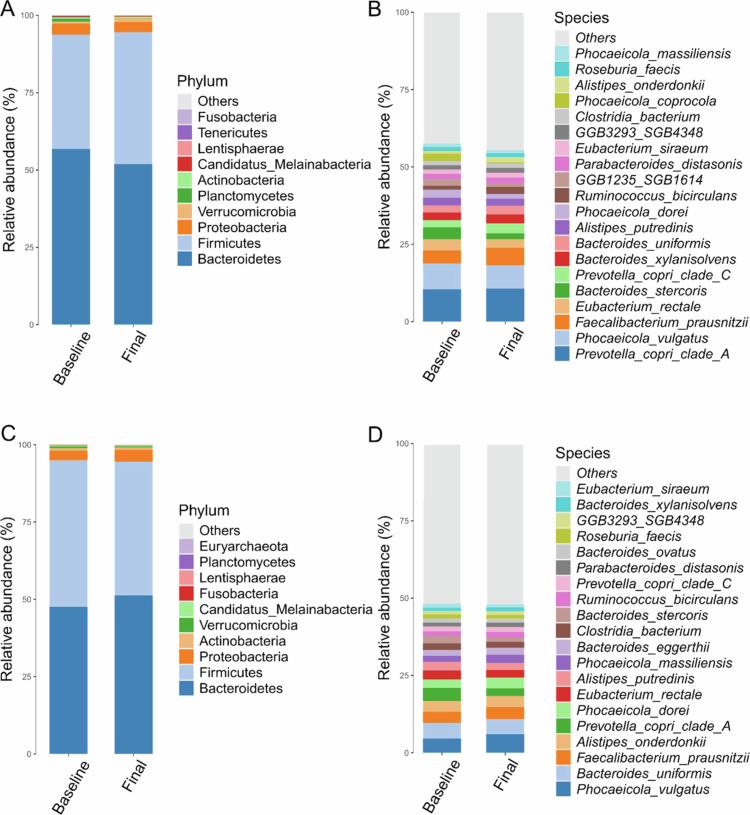
Taxonomic composition of microbial communities at the phylum (A and C) and species (B and D) levels. Stacked bar plots display the relative abundance of the top 10 phyla and top 20 species, with rare taxa grouped as “Others.” A and B = OEA group. C and D = Placebo group.

In the OEA group, the most represented species included *Prevotella copri* clade A and *Phocaeicola vulgatus*, followed by *Bacteroides stercoris*, *Faecalibacterium prausnitzii*, and *Eubacterium rectale* (Figure 3A−B). In contrast, in the placebo group, the top contributors included *P. vulgatus*, *Bacteroides uniformis, F. prausnitzii*, *P. copri* clade A, and *Alistipes onderdonkii* (Figure 3C−D). Overall, the relative abundance of major taxa remained broadly consistent between the baseline and final measurements in both groups, with no single species emerging as distinctly dominant.

#### Differential taxa and biomarkers

**Figure 4.**
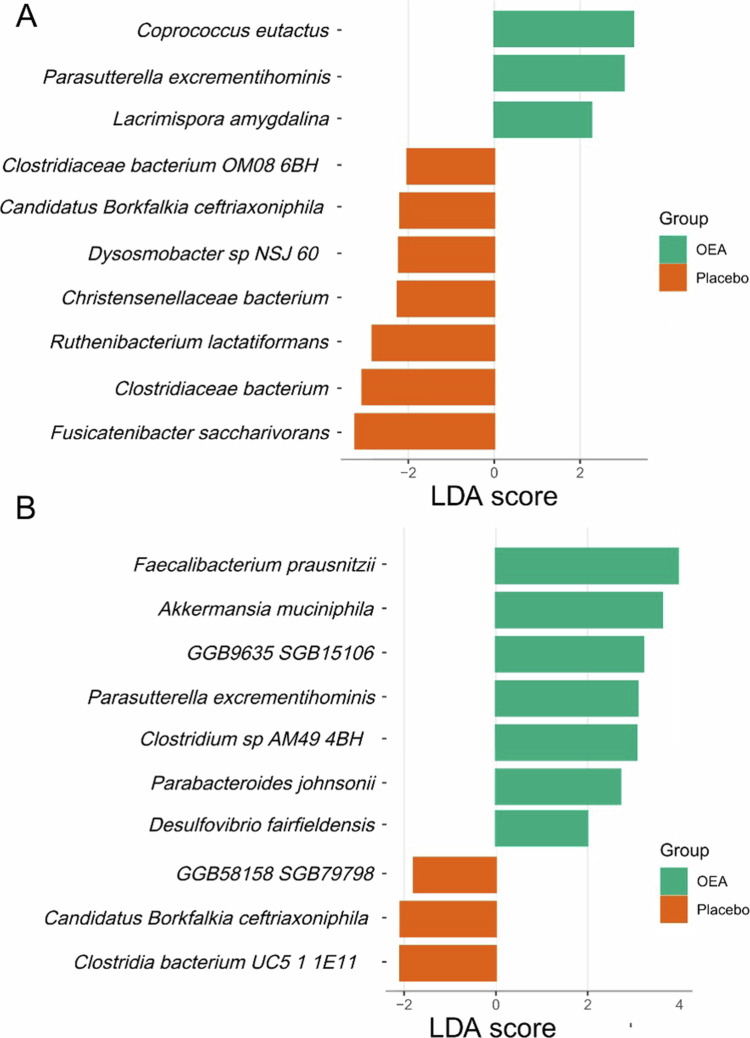
Biomarker species distinguishing OEA and placebo groups identified by Linear Discriminant Analysis Effect Size (LEfSe) at baseline (A) and at the final time point (B). Distinct sets of taxa were associated with each group at both time points, with several biomarkers unique to OEA and others unique to placebo.

OEA supplementation selectively enriched specific health-associated taxa, most notably Akkermansia muciniphila, despite no changes in overall diversity between groups (Figure 4A−B). Multivariate PERMANOVA revealed no significant differences in overall community structure between the baseline and final timepoints within either group. However, univariate Metastat analysis identified several bacterial taxa that were differentially abundant (*q* < 0.05) between baseline and final samples in the OEA group (Table 5).

Univariate Metastat analysis identified several bacterial taxa with pre-adjusted differences in abundance between baseline and final samples in the placebo group (*p* < 0.05). These differences did not remain significant in the placebo after FDR correction (*q* > 0.05). The specific taxa showing differential abundance differed between the two groups (Table 6).

**Table 5.** Microbiota with pre-adjusted and FDR-adjusted significant change from baseline to final in the OEA group.

*Taxa*	Mean baseline	Mean final	*p*-value	*q*-value
*GGB4599_SGB6362*	0	0.000161857	***	*
*Lawsonibacter* sp. *NSJ_52*	0	2.58E-05	***	*
*Faecalitalea cylindroides*	0	2.17E-05	***	*
*Candidatus Aristotella avistercoris*	0	1.36E-05	***	*
*Olsenella* sp. *GAM18*	0	1.22E-05	***	*
*Lachnoclostridium* sp. *An138*	0	8.89E-06	***	*
*Faecalimonas umbilicata*	7.17E-06	0	***	*
*Clostridia bacterium UC5 1 1E11*	5.22E-06	0	***	*
*GGB9239_SGB14181*	0	4.23E-06	***	*
*Streptococcus gordonii*	3.26E-06	0	***	*
*GGB9559_SGB14968*	0	4.23E-06	***	*
*Candidatus Schneewindia gallinarum*	0	3.04E-06	***	*
*Lactobacillus delbrueckii*	0	2.23E-06	***	*
*GGB2980_SGB3962*	0	2.21E-06	***	*
*GGB45432_SGB63101*	2.20E-06	0	***	*
*GGB52930_SGB73859*	7.58E-07	0	***	*
*GGB9532_SGB14933*	0	7.79E-07	***	*
*GGB9574_SGB14987*	0	6.08E-07	***	*
*Prevotella timonensis*	4.51E-07	0	***	*
*Candidatus Gallimonas intestinalis*	3.46E-07	0	***	*
*Lachnoclostridium edouardi*	0	4.19E-08	**	ns
*Clostridiaceae bacterium OM08 6BH*	0.000129099	0.000314342	*	ns
*Amedibacillus dolichus*	1.03E-06	9.39E-06	*	ns

*p < 0.05, **p < 0.01, ***p < 0.001, ns = not significant.

**Table 6.** Microbiota with pre-adjusted significant change from baseline to final in the Placebo group.

*Taxa*	Mean baseline	Mean final	*p*-value	*q*-value
*Lachnospira SGB5076*	0	0.000133456	***	ns
*Roseburia SGB4958*	5.49E-05	0	***	ns
*GGB9618_SGB15064*	2.04E-05	0	***	ns
*Prevotella timonensis*	0	1.19E-05	***	ns
*GGB3892_SGB5290*	4.10E-06	0	***	ns
*Actinomyces* sp. *S6 Spd3*	0	1.88E-06	***	ns
*Actinomyces* sp. *ICM47*	0	1.49E-06	***	ns
*Amedibacillus dolichus*	5.51E-07	0	***	ns
*Clostridiaceae* bacterium	0.004151	0.002372284	*	ns

*p < 0.05, ***p < 0.001, ns = not significant.

Linear discriminant analysis (LDA) identified distinct microbial signatures between groups at both baseline and week 12 (Figure 4A−B).

At Baseline, biomarker species for the OEA group included *Coprococcus eutactus*, *Parasutterella excrementihominis*, and *Lacrimispora amygdalina*, while the placebo group was characterized by *Clostridiaceae bacterium OM08_6BH*, *Candidatus Borkfalkia ceftriaxoniphila*, *Dysosmobacter sp. NSJ_60*, *Christensenellaceae bacterium*, *Ruthenibacterium lactatiformans*, *Clostridiaceae bacterium*, and *Fusicatenibacter saccharivorans* (Figure 4A).

Following 12 weeks of intervention, the microbial signatures shifted substantially. At week 12, biomarker species for the OEA group included *F. prausnitzii*, *A. muciniphila*, *GGB9635_SGB15106*, *P. excrementihominis*, *Clostridium* sp*. AM49_4BH*, *Parabacteroides johnsonii*, and *Desulfovibrio fairfieldensis,* while the placebo group was characterized by *GGB58158_SGB79798*, *Candidatus Borkfalkia ceftriaxoniphila*, and *Clostridia* bacterium *UC5_1_1E11* (Figure 4B).

To determine whether the between-group differences in biomarker species at week 12 reflected treatment-induced changes versus baseline heterogeneity, we performed mixed-effects modeling with time × treatment interaction for *A. muciniphila* and *F. prausnitzii*, two species of particular interest due to their well-established roles in metabolic health and gut barrier function.

*A. muciniphila* demonstrated a significant time x treatment interaction (F(1, 37.8) = 4.98, *p* = 0.032), indicating that changes in abundance over the 12-week intervention differed significantly between the OEA and placebo groups. Within-group paired analysis revealed that *A. muciniphila* abundance significantly increased in the OEA group from baseline to week 12 (mean change: +0.533%, 95% CI: 0.015 to 1.05, *p* = 0.016; paired *t*-test: t(19) = 2.017, *p* = 0.058), while decreasing in the placebo group (mean change: −0.14%, 95% CI: −0.426 to 0.139, *p* = 0.515; paired *t*−test: t(18) = −0.994, *p* = 0.333). Consequently, *A. muciniphila* relative abundance was significantly higher in the OEA group at week 12 after adjusting for baseline values (adjusted mean difference: 0.609%, 95% CI: 0.019 to 1.199, *p* = 0.043, ANCOVA).

*F. prausnitzii* showed a different pattern. While the time x treatment interaction did not reach statistical significance (F(1, 37.8) = 2.02, *p* = 0.163), within-group analysis demonstrated a significant increase in *F. prausnitzii* abundance in the OEA group (mean change: +1.31%, 95% CI: 0.186 to 2.431, *p* = 0.009; paired *t*−test: t(19) = 2.284, *p* = 0.034), whereas the placebo group showed no significant change (mean change: +0.35%, 95% CI: −0.422 to 1.127, *p* = 0.487; paired *t*−test: t(18) = 0.892, *p* = 0.384). At week 12, *F. prausnitzii* abundance was numerically higher in the OEA group after adjusting for baseline, though this difference approached but did not reach conventional statistical significance (adjusted mean difference: 1.242%, 95% CI: −0.093 to 2.578, *p* = 0.067, ANCOVA).

Additional taxa showing significant within-group changes from baseline to week 12 are summarized in Table 7.

**Table 7.** Biomarker species with significant changes from baseline to final in each group.

OEA Group	Function	Placebo Group	Function
*Akkermansia muciniphila*	Involved in mucus layer integrity, metabolic health, and immune regulation.^55^	*GGB58158 SGB79798*	Likely a metagenomic species bin (SGB = Species Genome Bin), meaning it represents a microbial species reconstructed from sequencing data but not necessarily well-characterized.
*Faecalibacterium prausnitzii*	A major producer of butyrate, linked to anti-inflammatory effects and gut health.^54^	*Candidatus Borkfalkia ceftriaxoniphila*	A candidate bacterial species potentially associated with antibiotic resistance (ceftriaxoniphila suggests a link to ceftriaxone, a common antibiotic).^56^
*GGB9635 SGB15106*	Likely an unnamed bacterial species identified through metagenomic sequencing.	*Clostridia* bacterium *UC5 1 1E11*	A member of the Clostridia class, which includes many gut-associated bacteria, some of which are involved in fermentation, short-chain fatty acid production, or pathobiosis (microbial imbalance in which potentially harmful (pathogenic) microorganisms become more dominant or active, often at the expense of beneficial microbes.^57^^,^^58^
*Parasutterella excrementihominis*	Linked to bile acid metabolism and gut homeostasis.^59^		
*Clostridium* sp. *AM49 4BH*	May have roles in metabolism, fermentation, or immune modulation.^60^		
*Parabacteroides johnsonii*	Associated with beneficial effects on metabolic conditions, including obesity and inflammatory bowel disease, due to their involvement in carbohydrate metabolism and production of short-chain fatty acids.^61^		
*Desulfovibrio fairfieldensis*	A sulfate-reducing bacterium that can produce hydrogen sulfide, which has potential effects on gut barrier function.^62^		

### Microbial metabolic functions

**Figure 5.**
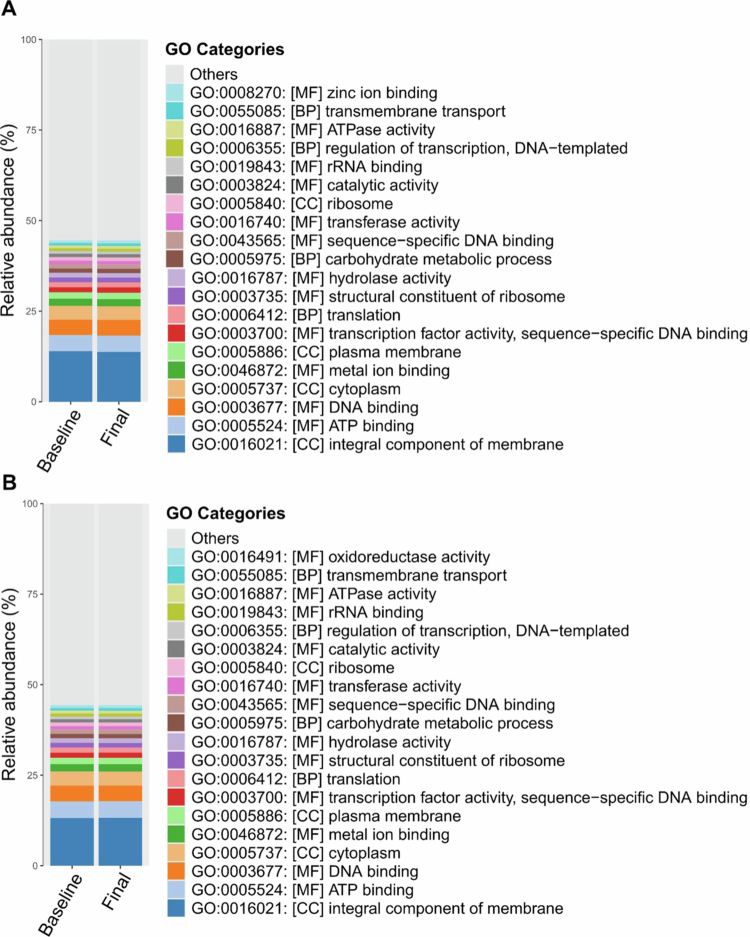
Distribution of top 20 Gene Ontology (GO) categories in Baseline and Final timepoint in the OEA group (A) and the Placebo group (B). Stacked bar charts represent the relative proportion of GO terms across major functional categories (biological process, molecular function, and cellular component).

Functional genes were grouped into GO terms to evaluate overall functional composition. Functional profiling of the microbiota revealed broadly stable microbial functional capacities between the baseline and final time points in both the OEA and placebo groups (Figure 5A−B). The relative distribution of the top functional categories remained consistent across time points, indicating no major shifts in dominant pathways.

### Differential pathways

Within-group Bray-Curtis distances based on functional profiles were significantly higher in the OEA group compared with placebo at both baseline and final time points, indicating greater inter-individual heterogeneity in microbial functions (Supplementary Fig. S2). While no significant differences were observed in overall functional profiles between groups based on multivariate analysis, univariate analyzes identified several individual functions showing pre-adjusted between-group differences (*p* < 0.05), however, these did not remain significant following FDR correction. Within each group, overall functional profiles remained stable from baseline to final. No pre-adjusted changes were observed in the placebo group, whereas the OEA group exhibited pre-adjusted alterations in the abundance of several functional groups from baseline to the final time point (Table 8). Seven predicted functional pathways showed increased relative abundance (*p* < 0.05), including those involving 4-hydroxybenzoyl-CoA reductase, glutamyl-tRNA reductase, gluconate 5-dehydrogenase, hydroxymethylbilane synthase, *N*-acylglucosamine-6-phosphate 2-epimerase, acetylornithine deacetylase, and carbamate kinase, whereas the pathway involving telomere maintenance was reduced.

**Table 8.** Differential Functional Pathways in the OEA group identified with Metastat paired analysis (*p* < 0.05).

	Relative Abundance (%)			
Taxa	Baseline	Final	*p*-value	*q*-value
4-hydroxybenzoyl-coa reductase activity	0.000010	0.000017	**	ns
Glutamyl-trna reductase activity	0.000011	0.000016	*	ns
Gluconate 5-dehydrogenase activity	0.000010	0.000014	*	ns
Telomere maintenance	0.000086	0.000069	*	ns
Hydroxymethylbilane synthase activity	0.000012	0.000017	*	ns
*N*-acylglucosamine-6-phosphate 2-epimerase activity	0.000009	0.000012	*	ns
Acetylornithine deacetylase activity	0.000008	0.000014	*	ns
Carbamate kinase activity	0.000022	0.000029	*	ns

*p < 0.05, **p < 0.01, ns = not significant.

### SCFAs and calprotectin

SCFA results showed that there were no significant differences between groups or changes from baseline to week 12 within each group (Table 9).

**Table 9.** Fecal short chain fatty acid and calprotectin levels'.

	OEA				Placebo				
	SCFA/g dry weight		SCFA (%)		SCFA/g dry weight		SCFA (%)		*p*-value
	Baseline	Week 12	Baseline	Week 12	Baseline	Week 12	Baseline	Week 12	0.37
Acetic Acid	39.34 (21.14)	51.44 (77.11)	60.78 (5.44)	60.44 (10.05)	43.45 (16.23)	39.67 (13.14)	62.55 (9.2)	60.99 (9.38)	0.49
Propionic Acid	10.6 (5.93)	11.77 (9.39)	16.5 (5.04)	17.58 (5.61)	11.16 (6.73)	10.5 (5.97)	14.62 (4.84)	15.33 (5.22)	0.28
IsoButyric Acid	1.01 (0.7)	1.21 (1.64)	1.64 (0.77)	1.84 (1.37)	1.73 (0.96)	1.48 (0.79)	2.32 (0.61)	2.34 (1.04)	0.48
Butyric Acid	10.08 (6.51)	12.98 (21.98)	15.32 (4.51)	14.19 (5.49)	9.8 (5.77)	9.44 (5.19)	12.53 (4.26)	13.63 (4.28)	0.26
Isovaleric Acid	1.4 (1.05)	1.56 (1.98)	2.25 (1.19)	2.48 (1.92)	2.66 (1.56)	2.18 (1.34)	3.57 (1.09)	3.5 (1.84)	0.28
Valeric Acid	1.39 (0.95)	1.79 (2.61)	2.18 (0.83)	2.39 (1.14)	2.3 (1.82)	2.05 (1.82)	2.92 (1.21)	3.06 (2.2)	0.32
Hexanoic Acid	0.61 (0.77)	1.01 (3.05)	1.01 (1.14)	0.84 (0.93)	0.74 (0.94)	0.54 (0.5)	1.05 (1.04)	0.87 (0.77)	0.48
Heptanoic Acid	0.19 (0.3)	0.2 (0.42)	0.32 (0.5)	0.25 (0.25)	0.24 (0.3)	0.18 (0.15)	0.44 (0.66)	0.3 (0.25)	0.97
Calprotectin (ug/g)	0.1 (0.1)	0.13 (0.19)	−	−	0.55 (1.38)	0.6 (1.71)	−	−	0.37

Data is presented as mean (SD). *p*-values presented are for the change from baseline values.

### Fibroscan (liver)

There were no significant differences between groups or between baseline and week 12 values from the liver safety scans (Table 10).

**Table 10.** Liver scan measurements and clinical parameters.

	OEA			Placebo			
	Baseline	Week 12	Change	Baseline	Week 12	Change	*p*-Value
Liver size (mm)	127.35 (25.63)	125.1 (26.19)	−3.62 (28.37)	133.52 (24.57)	125.85 (25.34)	−5.52 (28.52)	0.83
Portal vein measure (mm)	10.72 (1.64)	11.08 (1.76)	0.45 (2.1)	11.46 (1.58)	10.93 (2.09)	−0.69 (2.24)	0.09
Hepatoportal flow with Doppler velocity (cm/s)	23.92 (9.8)	22.29 (6.91)	−0.35 (13.45)	19.93 (5.15)	20.76 (4.67)	1.7 (6.21)	0.52
Median liver stiffness value (kPa)	5.46 (1.17)	5.67 (1.93)	0.15 (1.15)	5.92 (3.4)	5.13 (0.73)	−0.08 (1.12)	0.51
Median velocity (m/s)	1.33 (0.12)	1.5 (0.83)	0.17 (0.8)	1.36 (0.3)	1.3 (0.09)	−0.01 (0.13)	0.33

Data is presented as mean (SD). *p*-values presented are for the change from baseline values.

### Questionnaires

There were no significant differences between groups or between baseline and week 12 for the results from any of the questionnaires (data not shown).

## Discussion

This study provides new insights into the effects of OEA supplementation on systemic inflammation, intestinal barrier function, and gut microbiota composition in humans. Our results demonstrate that OEA significantly modulates both immunological markers and microbial profiles compared with placebo over a 12-week period. The observed increases in occludin (OCLN) and interleukin-2 (IL-2), along with reductions in interleukin-1β (IL-1β), suggest that OEA may enhance intestinal barrier integrity and modulate inflammatory responses. These findings align with previous research indicating that OEA can influence tight junction proteins and cytokine profiles, primarily through activation of PPAR-*α* and related signaling within the broader endocannabinoidome, rather than via direct CB1/CB2 receptor activity.^13^

Endogenous OEA production is reduced in states of overweight and obesity, likely due to impaired intestinal lipid signaling and dysregulated endocannabinoid-related pathways.^41^^,^^56^ This deficiency may explain the positive response from overweight and obese individuals to OEA supplementation, which can restore pathway signaling and promote metabolic homeostasis. This is supported by our finding that in participants with a BMI < 35, OEA supplementation led to a significant reduction in body weight compared with placebo. Although this was not observed in the overall cohort, these subgroup findings align with previous reports of OEA promoting satiety and reducing body weight through activation of PPAR-*α* and gut–brain signaling pathways.^57^^,^^58^ This suggests that individuals with moderate obesity may be more responsive to OEA’s weight-regulatory effects than those with more severe obesity.

The observed modulation of occludin (OCLN), IL-2 and IL-1 *β* in the OEA group suggests that OEA may influence pathways relevant to intestinal barrier regulation and immune signaling. Circulating OCLN has been proposed as a proxy marker of tight-junction turnover and epithelial remodeling, with elevated serum levels reported in conditions associated with altered gut barrier dynamics rather than direct measures of junctional integrity.^59^^,^^60^ Although circulating OCLN does not reflect mucosal expression per se, increased levels are generally interpreted as indicating enhanced tight junction shedding or turnover, which may occur during both barrier disruption and adaptive epithelial renewal. In this context the Week-12 increase in circulating OCLN observed in the OEA group may reflect altered tight-junction dynamics rather than a direct improvement in barrier integrity. This interpretation is consistent with preclinical studies showing that OEA modulates intestinal permeability and epithelial homeostasis in models of metabolic endotoxemia and colitis.^39^^,^^61^ However, direct assessment of intestinal permeability or mucosal tight-junction expression would be required to confirm whether these circulating biomarker changes translate into functional improvements in gut barrier stability.

The lack of significant changes in zonulin, claudin-1, and mucin-2 suggests that OEA supplementation did not broadly affect mucosal defense or paracellular permeability. However, the observed upregulation of occludin indicates a specific enhancement of tight junction integrity. This selective effect suggests that OEA may strengthen gut barrier function without disrupting the overall stability of the intestinal environment.

In contrast to OCLN, the cytokine responses to OEA appeared transient. IL-2 increased at Week 6 while IL-1β decreased at the same time point; however, neither effect persisted to Week 12. These temporal patterns may reflect an early immune-modulatory response to OEA, consistent with the notion that cytokine networks adapt dynamically during the onset of epithelial or metabolic changes. IL-1β is a key mediator of chronic inflammation and disrupts tight-junction integrity via NF-κB and MLCK pathways^62–64^; thus, its transient reduction may contribute to short-term improvements in epithelial function. Likewise, the early rise in IL-2, a cytokine involved in T‑cell proliferation and mucosal immune balance, may indicate enhanced immune regulatory signaling during the initial supplementation phase.^65^^,^^66^ This supports a potential immunoregulatory role of OEA, although its reliability as a biomarker has been variable in human studies.^67^ The absence of sustained changes at Week 12 suggests that these immune effects may be temporally limited or subject to feedback regulation. Taken together, these cytokine changes suggest that OEA could potentially influence gut health and metabolic regulation by modulating immune responses. Notably, levels of other inflammatory and metabolic markers, including TNF-*α*, IFN-*γ*, GST, and endotoxin, did not differ significantly between groups, indicating that OEA’s effects may be specific rather than act broadly as an anti-inflammatory.

While alpha diversity and overall microbiota richness did not significantly differ between groups, the OEA group exhibited notable changes in microbial composition and functional potential. At baseline, the gut microbiota in the OEA group was characterized by greater within-group variability and dominance of *P. copri* clade A and *P. vulgatus*. These taxa are commonly associated with carbohydrate metabolism^68^^,^^69^ and are considered beneficial for maintaining intestinal barrier integrity.^70^ However, their roles appear context-dependent, as they have also been linked to both protective and pro-inflammatory effects depending on host physiology.^68^^,^^71^ Although diversity indices remained unchanged over time, univariate analyzes revealed dynamic shifts in the relative abundance of several microbial taxa and biomarker species, particularly in the OEA group.

One of the most notable findings of this study was the differential modulation of key health-promoting bacterial species by OEA supplementation. Notably, species-level analysis identified key taxa that distinguished the OEA group from the placebo group, including *F. prausnitzii*, *A. muciniphila*. Specifically, OEA treatment resulted in significant enrichment of *A. muciniphila*, a mucin-degrading bacterium increasingly recognized as a next-generation probiotic with potent metabolic benefits. The significant time × treatment interaction (*p* = 0.032) and substantial within-group increase in *A. muciniphila* abundance (+0.533%, *p* = 0.016) demonstrate that OEA selectively promotes this beneficial species. Additionally, *F. prausnitzii*, one of the most abundant butyrate-producing bacteria in the healthy human gut, showed significant enrichment in the OEA group (+1.308%, *p* = 0.009). While the time × treatment interaction was not statistically significant, the robust within-group increase in OEA-treated participants, coupled with the absence of change in placebo, suggests a beneficial modulation of the gut ecosystem.

Both *F. prausnitzii* and *A. muciniphila* are recognized as beneficial microbes with established roles in intestinal and metabolic health, particularly for their anti-inflammatory properties and contributions to maintaining mucosal integrity.^72^^,^^73^ Reduced *A. muciniphila* abundance has been consistently reported in obesity and metabolic dysfunction, while human supplementation studies demonstrate improvements in insulin sensitivity, inflammation, and cardiometabolic risk markers, positioning this species as a clinically relevant microbiome target.^45^^,^^74^ *F. prausnitzii* is a major butyrate producer, and its abundance is consistently associated with anti-inflammatory activity and improved gut barrier function.^72^^,^^75^ Reduced levels of *F. prausnitzii* have been linked to inflammatory bowel disease and metabolic syndrome, while restoration is associated with improved mucosal tolerance.^75^ Similarly, *A. muciniphila* resides in the mucus layer and contributes to mucosal integrity, glucose metabolism, and host immune regulation.^74^^,^^76^ Clinical studies have shown that supplementation with pasteurized *A. muciniphila* improves insulin sensitivity and reduces markers of systemic inflammation in overweight and obese adults.^45^ The higher relative abundance of these taxa in the OEA group may therefore reflect a shift toward a more eubiotic and metabolically favorable microbial profile. In contrast, the placebo group showed no significant changes in microbial composition or immune markers, suggesting that the observed effects were specific to OEA.

This finding is particularly relevant given that *A. muciniphila* has been consistently associated with improved glucose homeostasis, reduced inflammation, enhanced gut barrier function, and protection against obesity-related metabolic dysfunction.^77^ The concurrent decrease in *A. muciniphila* in the placebo group suggests that OEA may protect against natural decline in this species during caloric modulation or aging.^78^

Although alpha and beta diversity did not differ significantly between groups, functional metagenomic analysis revealed distinct microbial functional alterations in the OEA group. Several enzymatic functions were significantly enriched, providing insights into potential mechanisms underlying the physiological benefits of OEA. These included 4-hydroxybenzoyl-CoA reductase, glutamyl-tRNA reductase, gluconate 5-dehydrogenase, hydroxymethylbilane synthase, *N*-acylglucosamine-6-phosphate 2-epimerase, acetylornithine deacetylase, and carbamate kinase. In contrast, telomere maintenance activity was significantly depleted. These findings are consistent with previous studies reporting functional shifts in gut microbiota following dietary or pharmacological interventions.^79^^,^^80^

Among the pathways showing pre-adjusted changes was the higher relative abundance of 4-hydroxybenzoyl-CoA reductase, an enzyme involved in the degradation of aromatic compounds such as phenolics.^81^ This function likely contributes to enhanced microbial detoxification and energy harvesting from dietary components, potentially leading to a less pro-inflammatory gut environment.^82^ Similarly, the enrichment of glutamyl-tRNA reductase and hydroxymethylbilane synthase, both involved in tetrapyrrole and heme biosynthesis pathways, suggests enhanced microbial metabolic capacity and redox regulation, which may support improved microbial resilience and host-microbiota redox balance, potentially contributing to mucosal protection and metabolic homeostasis.^83^^,^^84^

The observed pre-adjusted increase in gluconate 5-dehydrogenase activity, a key enzyme in gluconate metabolism and oxidative energy pathways, suggests enhanced microbial capacity for generating NADPH, an essential cofactor for antioxidant defense.^85^^,^^86^ This may be particularly important for mitigating oxidative stress in the gut and preserving epithelial barrier integrity. In parallel, the enrichment of *N*-acylglucosamine-6-phosphate 2-epimerase suggests an enhanced microbial capacity for sialic acid biosynthesis.^87^ Sialic acids are essential for cell surface modifications, bacterial communication, and gut colonization.^88^^,^^89^ Such functional enrichment may facilitate beneficial bacterial interactions and support mucosal integrity, thereby contributing to anti-inflammatory and metabolic effects.^89^

Additionally, the enrichment of acetylornithine deacetylase suggests altered microbial amino acid metabolism, specifically in the arginine pathway, which contributes to nitrogen balance and membrane stability.^90^^,^^91^ Likewise, the higher relative abundance of carbamate kinase, an enzyme involved in energy metabolism, suggests a more metabolically active microbiome with the potential to sustain enhanced biosynthetic and protective functions.^92^

In contrast, telomere maintenance activity was found to be depleted, which may reflect reduced microbial replication stress or a shift toward a more stable and less proliferative microbial population.^93^ This could be indicative of a more balanced ecosystem established through OEA supplementation, favouring functional efficiency over excessive microbial turnover. Despite pre-adjusted differences observed in certain pathways, the absence of FDR–adjusted significance indicates the interpretation of these functional pathways remains speculative.

Collectively, these functional shifts support the hypothesis that OEA not only modulates host metabolic and immune responses but also selectively enhances microbial functions that promote gut homeostasis, epithelial protection, and metabolic regulation. Importantly, the absence of significant changes in overall functional diversity, despite these targeted pathway alterations, highlights the complexity of microbiome-host interactions. This suggests that OEA may exert its physiological effects not through broad restructuring of the microbiome, but via precise modulation of specific microbial functions with direct relevance to host health. Further studies are warranted to elucidate how these microbial activities interact with host signaling networks to mediate the systemic effects of OEA.

The selection of a 250 mg/day dose of OEA in the present trial is supported by prior human research and studies we have previously conducted using the same lipid-amide delivery technology. Multiple clinical investigations have demonstrated that daily oral OEA at 250 mg for 8–12 weeks is safe and associated with favorable effects on metabolic and inflammatory markers in overweight and obese populations,^94–96^ establishing this dose as biologically active and well tolerated. In addition, our earlier work examining the structurally related fatty-acid amide palmitoylethanolamide (PEA; Levagen+) formulated with LipiSperse® showed significantly enhanced oral absorption and excellent tolerability compared with standard formulations, providing a strong rationale for applying the same delivery approach to OEA here.^97^^,^^98^ Taken together, the existing literature and our prior research support 250 mg/day as a justified therapeutic dose that balances safety with the potential for meaningful biological effects in humans.

While this study assessed the gut microbiome and biomarkers, it was not specifically designed to evaluate causality between microbiota changes and biomarker responses. As such, the microbiome and biomarker results are presented as independent observations (no correlation conducted). The observed shifts in specific microbiome taxa and predicted functional pathways should therefore be interpreted separately, rather than as direct drivers of the other outcome measures. Further studies incorporating dedicated analyzes or targeted pathways would help to clarify potential microbiota interactions in the context of OEA supplementation.

While the present study was not powered for formal mediation or microbiota–host correlation analyzes, exploratory assessments indicated modest, non-significant associations between key OEA-associated taxa and host biomarkers. These preliminary findings suggest that microbial changes may accompany, rather than directly mediate, the physiological effects of OEA. Additionally, OEA concentrations were not quantified in plasma or fecal samples in the present study, as the trial was not designed as a pharmacokinetic assessment. However, prior human trials have shown that oral OEA can exert metabolic, inflammatory, and microbiota‑related effects without requiring substantial increases in circulating OEA levels, consistent with its predominantly local actions within the intestinal mucosa and enteric signaling pathways.

## Conclusion

This study showed 12 weeks of OEA supplementation in adults with obesity selectively modulates the gut microbiome and intestinal barrier function without changing overall microbial diversity. OEA enriched beneficial species, particularly *Akkermansia muciniphila* and *Faecalibacterium prausnitzii*, and increased circulating occludin, suggesting improved tight-junction integrity, alongside transient immune modulation. Functional metagenomics indicated enhanced antioxidant, redox, and amino acid metabolism pathways, supporting targeted improvements in gut homeostasis rather than broad microbial shifts. These effects were specific, with most inflammatory and metabolic markers unchanged, highlighting a focused mechanism of action. The enrichment of mucin-degrading and butyrate-producing taxa and improved barrier markers suggest OEA may help address obesity-related metabolic dysfunction via microbiome-targeted pathways. Future studies should assess intestinal permeability directly, clarify mechanisms, and explore dose–response relationships and individual variability to optimize therapeutic use.

## Supplementary Material

Picture_S2.jpg Supplemental Material

Picture_S1.jpg Supplemental Material

Supplementary Material Supplemental Material

## Data Availability

The datasets generated and/or analyzed during the current study are not publicly available due to commercial interests but are available from the corresponding author on reasonable request.
