# Supplementary figures and images for: Oleoylethanolamide supplementation enriches Akkermansia muciniphila and modulates intestinal barrier function in adults with obesity: A randomized, double-blind, placebo-controlled trial
Source: Gut Microbes Rep. 2026 Feb 21;3(1):2622259. doi: 10.1080/29933935.2026.2622259 (PMC13037523; doi:10.1080/29933935.2026.2622259)

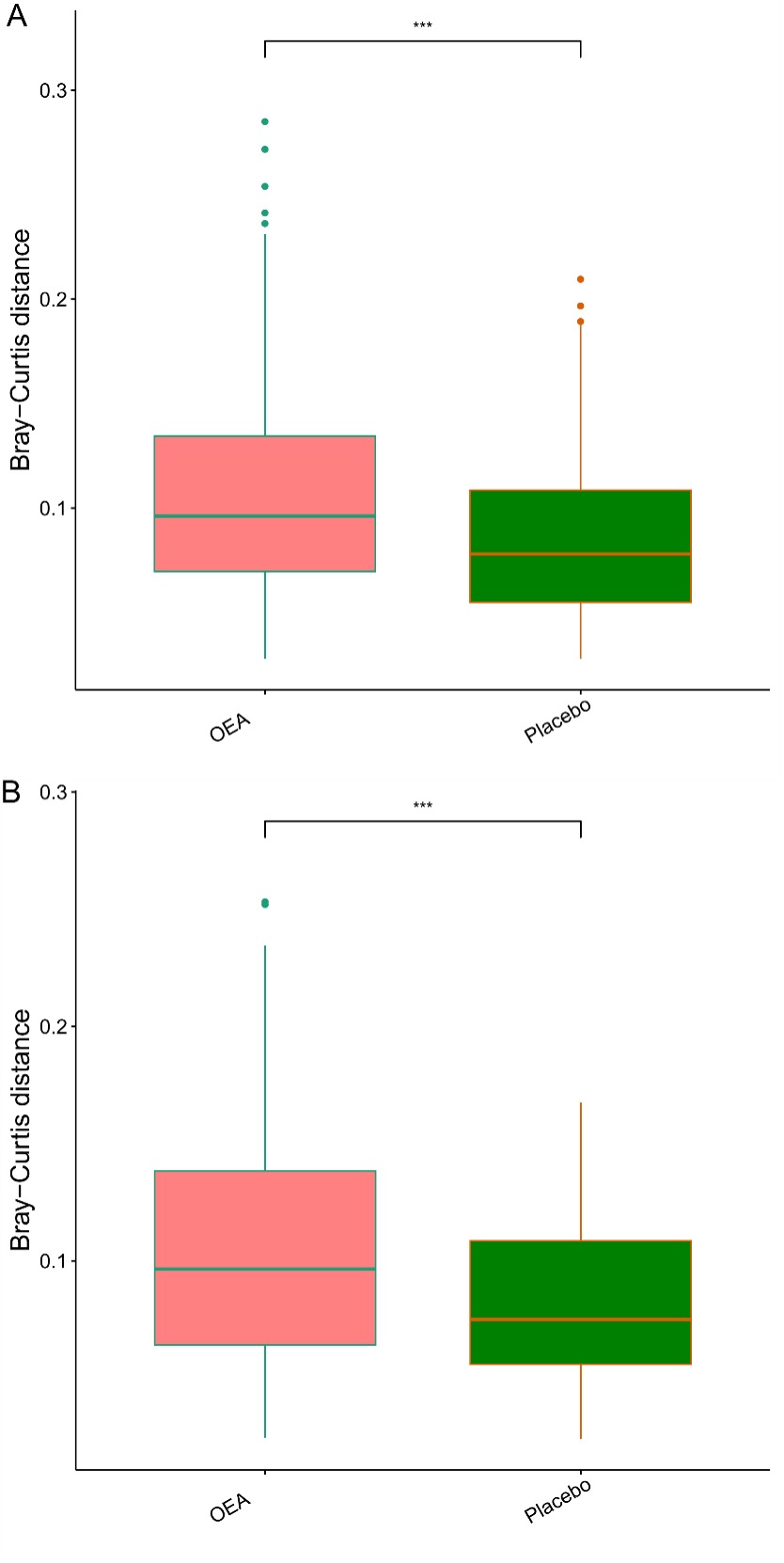

Supplement: Picture_S2.jpg — Supplemental Material [file KGMR_A_2622259_SM5080.jpg]

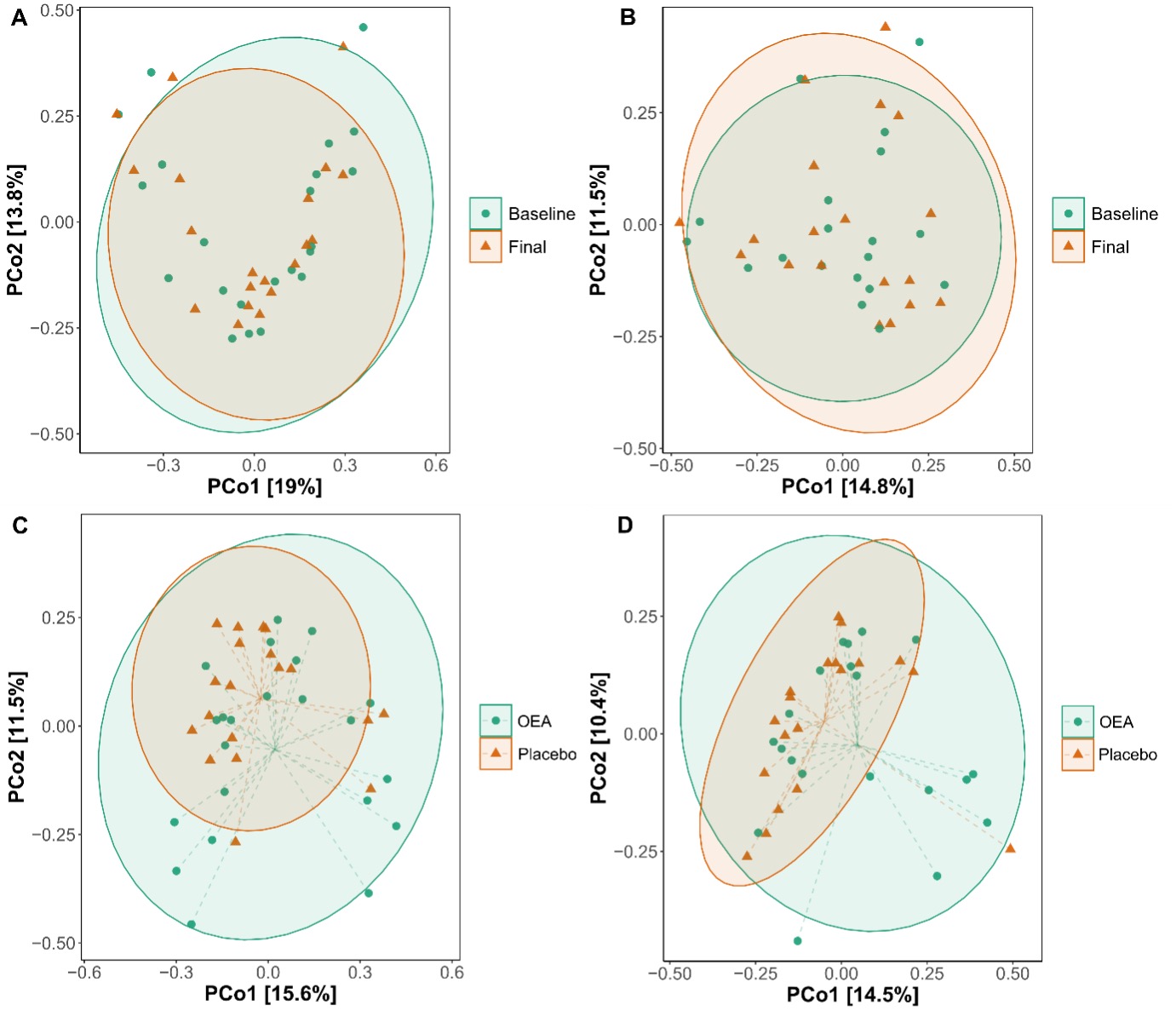

Supplement: Picture_S1.jpg — Supplemental Material [file KGMR_A_2622259_SM5079.jpg]
