# Supplementary material for: Oleoylethanolamide supplementation enriches Akkermansia muciniphila and modulates intestinal barrier function in adults with obesity: A randomized, double-blind, placebo-controlled trial
Source: Gut Microbes Rep. 2026 Feb 21;3(1):2622259. doi: 10.1080/29933935.2026.2622259 (PMC13037523; doi:10.1080/29933935.2026.2622259)
Supplement: Supplementary Material — Supplemental Material [file KGMR_A_2622259_SM5252.docx]

**Supplementary Figure S1.** Principal coordinates analysis (PCoA) of Bray-Curtis distances showing microbial community composition across study groups. A) baseline vs. final time point within the OEA group, B) baseline vs. final time point within the placebo group, C) OEA vs. placebo at baseline, and D) OEA vs. placebo at the final time point. Each point represents an individual sample.

**Supplementary Figure S2.** Within-group Bray–Curtis distances based on microbial functional profiles at baseline and final time points in the OEA and placebo groups. Higher distances indicate greater inter-individual variability in functional potential, whereas lower distances reflect more homogeneous functional profiles.
